# Rupture-mediated large uterine defect at 30th gestational week with protruded amniotic sac and fetal head without fetal compromise after laparoscopic electromyolysis: Case report and literature review

**DOI:** 10.1097/MD.0000000000032221

**Published:** 2022-12-23

**Authors:** Seung-Woo Yang, Sang-Hee Yoon, Jin-Sung Yuk, Kyoung-Chul Chun, Myeong Ja Jeong, Myounghwan Kim

**Affiliations:** a Department of Obstetrics and Gynecology, Sanggye Paik Hospital, School of Medicine, Inje University, Seoul, Republic of South Korea; b Department of Obstetrics and Gynecology, Inje University College of Medicine, Ilsan-Paik Hospital, Gyeonggi, South Korea; c Department of Radiology, Sanggye Paik Hospital, School of Medicine, Inje University, Seoul, Republic of South Korea.

**Keywords:** fetal compromise, fetal morbidity, laparoscopic electomyolysis, pregnancy, uterine fibroids, uterine rupture

## Abstract

We describe a case of a rupture–mediated large uterine defect, which occurred on the 30th gestation week presenting a protruding amniotic sac sac without fetal compromise after a laparoscopic electromyolysis.

**Case presentation:**

A 28-year-old woman in her 30^th^ week of gestation (gravida 2, para 0) presented with whole abdominal and right lower quadrant pain at Sanggye Paik Hospital. Ultrasound examination showed normal amniotic fluid and placentation but with breech presentation. She had undergone laparoscopic right ovarian cystectomy due to endometriosis 5 years earlier. Cardiotocography revealed an intermittent variable deceleration and no uterine contraction. Magnetic resonance imaging ruled out acute appendicitis. Four hours later, we observed a protrusion of the amniotic sac with the fetal head through a large uterine defect on magnetic resonance imaging, and performed emergency cesarean section. A boy was delivered without fetal compromise. During the cesarean section, multiple myometric wall defects and thinning were identified. After reconstruction of the uterine wall, the flaccid uterus bled persistently; thus, a cesarean hysterectomy was performed. Packed red cells and frozen plasma were transfused. The mother and neonate had uneventful puerperal and neonatal courses, respectively. After cesarean hysterectomy, we were informed that the mother had undergone a combined laparoscopic electromyolysis during the laparoscopic right ovarian cystectomy. Three years later, the child showed normal neural development.

**Conclusions:**

Before myomectomy or electromyolysis, patients should be informed of the possibility of uterine rupture during subsequent pregnancies. If a pregnant woman has abdominal pain, clinicians should take a detailed history of uterine surgery and consider uterine rupture. Although, fortunately, the outcomes in this case were uneventful, urgent delivery is required when uterine rupture is diagnosed.

## 1. Introduction

Uterine rupture during pregnancy can be life threatening. Both the mother and fetus are at risk of serious morbidities and mortality. Most uterine ruptures occur in patients who have undergone previous transmyometrial surgical incisions, typically for a cesarean section or a myomectomy. Uterine rupture has been associated with the presence of a previous cesarean delivery, as the latter has been found to precede a uterine rupture in 92% of the cases.^[1]^ The risk of uterine rupture is approximately 0.3% in patients with previous lower uterine segment cesarean section, whereas it is approximately 4% to 9% after a classical cesarean delivery.^[2]^

The occurrence frequency of uterine rupture after laparoscopic myomectomy has been reported to be < 1%, but its frequency after myolysis is unclear.^[3]^ Uterine rupture may be incomplete or complete; in an incomplete rupture, the uterine serosa remains intact, whereas a complete rupture bears a full-thickness separation of the uterine wall and expulsion of the fetus and/or the placenta into the abdominal cavity. A complete rupture is usually more serious than an incomplete 1 and possibly life threatening for both the fetus and the mother.^[4]^ Uterine ruptures with defects large enough to cause fetal head protrusions have resulted in hypoxic ischemic encephalopathies, severe asphyxia, and even fetal deaths.^[5,6]^

Surgical intervention within 10 to 37 minutes of uterine rupture is essential to minimize the risk of permanent perinatal fetal injury.^[7–9]^ However, even if delivery occurs within this time frame, the development of serious neonatal abnormalities such as severe hypoxia or metabolic acidosis are still at risk of occurring.

We report a fortunate and rare case of uterine rupture during pregnancy that caused a uterine defect, large enough to allow the fetal head and the amniotic sac to protrude. Nevertheless, surgery eventuated in the successful delivery of a healthy child, albeit with a long time-to-delivery interval.

Given the anonymous nature of this case report, it has been exempted from review by the Review Board of our Institute.

## 2. Case presentation

A 28-year-old woman on her 30^th^ week of gestation (gravida 2, para 0) was presented at the Sanggye Paik Hospital with whole abdominal and right lower quadrant pain via a local obstetric clinic.

The abdomen was distended because the uterus was enlarged at 30-week gestation. A physical exam showed no abdominal or rebound tenderness. Laboratory findings showed: hemoglobin 13.1 g/dL, white blood cells 10,600/*µ*L, platelets 266,000/*µ*L, and C-reative protein < 0.3 mg/dL. Urinary analysis was negative for nitrites and red blood cells.

**Figure 1.**
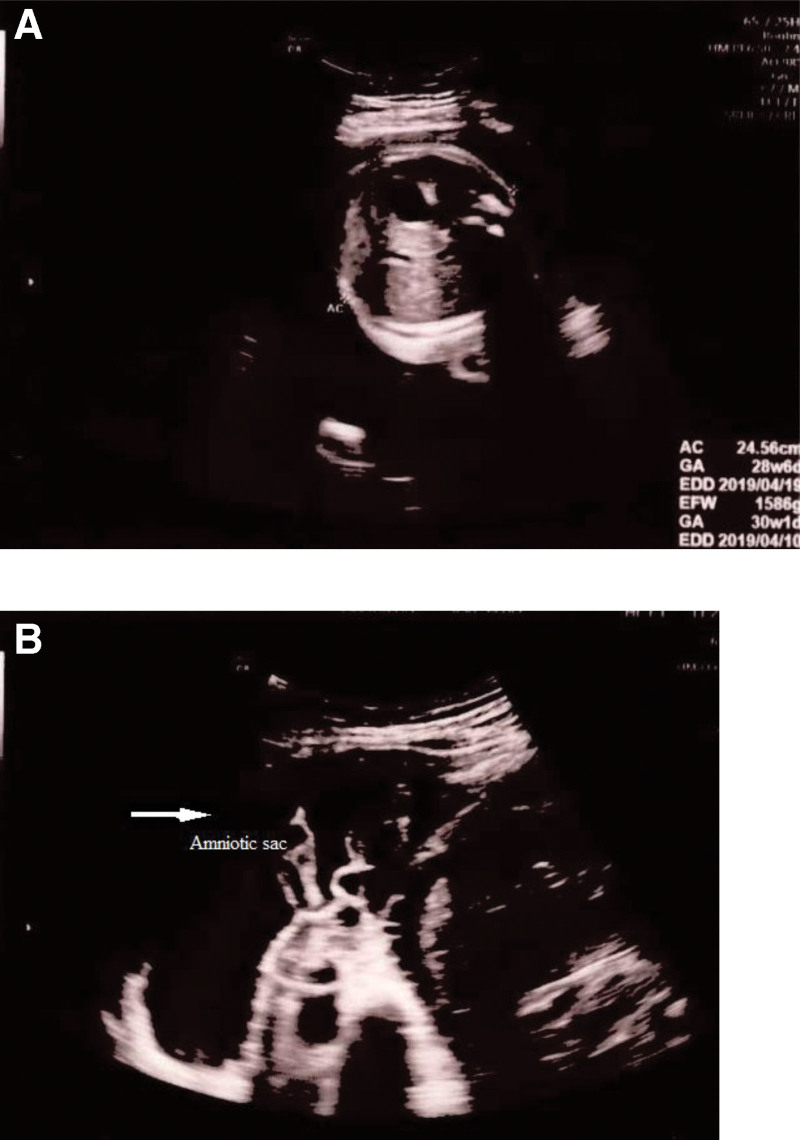
Ultrasound examination before and after uterine rupture. (a) Absence of abnormal findings related to amniotic fluid volume and placentation. (b) Protruding amnionic sac (arrow) observed through a large uterine defect.

**Figure 2.**
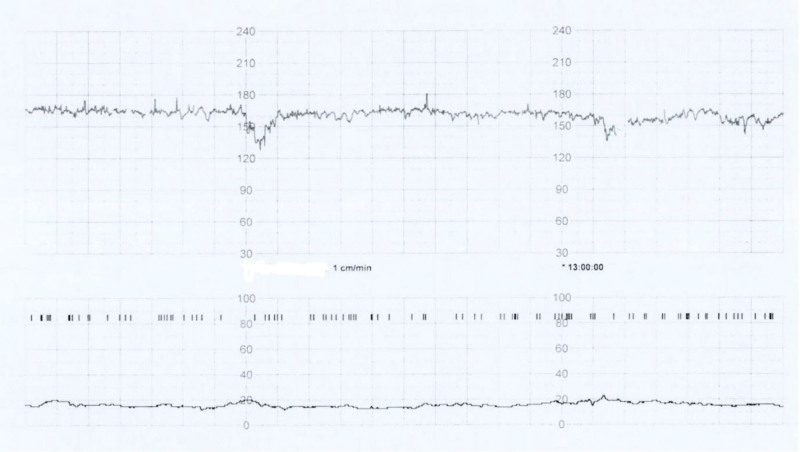
Cardiotocographic monitoring indicating an intermittent variable deceleration and absence of uterine contractions.

**Figure 3.**
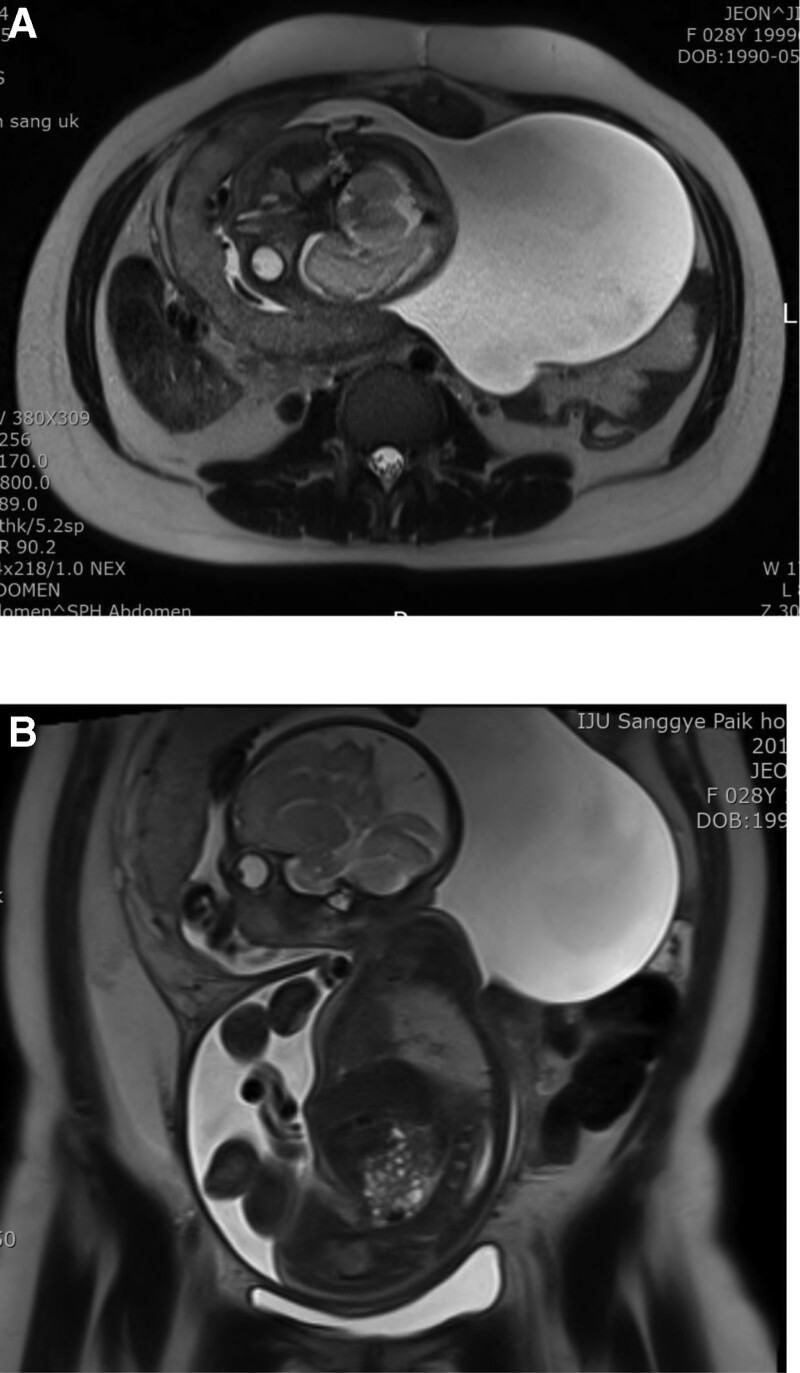
Magnetic resonance imaging of patient before and after uterine rupture. (a) Axial view showing no evidence of appendicitis. (b) Several hours later, coronal view shows the fetal head and the amniotic sac protruding through a large uterine defect.

**Figure 4.**
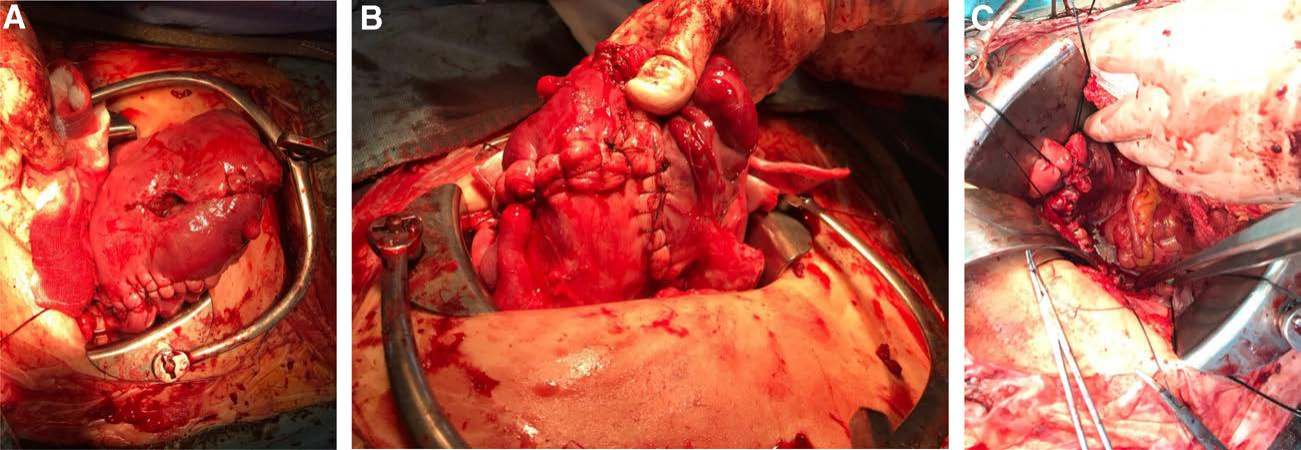
Multiple myometrial uterine wall defects and thinning were identified during a cesarean section. (a) Anterior wall of uterus. (b) Posterior wall of the uterus. (c) Abdominal space after cesaeran hysterectomy.

Ultrasound examination identified breech presentation, an amniotic fluid index of 27.18 cm, placentation in the anterior uterine wall, and an estimated fetal weight of 1580 g (Fig. 1A). The mother had undergone a laparoscopic right ovarian cystectomy due to endometriosis 5 years earlier. Cardiotocographic monitoring revealed an intermittent variable deceleration, a reassuring fetal heart rate pattern, and the absence of uterine contraction (Fig. 2). Magnetic resonance imaging (MRI) performed in the Department of General Surgery of our institution ruled out acute appendicitis. Several hours later, we noticed that the amniotic sac was protruding through a large uterine defect (Fig. 3), and ultrasound examination also showed protrusion of the amniotic sac (Fig. 1B). The patient was subjected to an emergency cesarean section, which enabled the delivery of a male neonate (1860 g and 40 cm) without fetal compromise. His Apgar scores at 1 minute, 5 minutes, and 10 minutes were 4, 6 and 8, respectively, and an umbilical arterial gas analysis indicated a pH value of 7.253, a partial pressure of oxygen of 43.4 mm Hg, a partial pressure of carbon dioxide of 48.6 mm Hg, an ${HCO}_{3}^{-}$ concentration of 21.5 mmol/L, a saturation of oxygen of 88.1%, and a base excess of -5.7 mmol/L. During the cesarean section, multiple myometrial defects and thinning of the uterine wall were uncovered. Notably, these myometrial wall defects extended anteroposteriorly via the uterine fundus to the left uterine wall (Fig. 4A and B), and a uterine wall reconstruction did not suffice to restrain the bleeding from the flaccid uterus (Fig. 4B). A pulse rate of 140 to 150 beat per minute also persisted. Cesarean hysterectomy was performed (Fig. 4C). Packed red cells of 5 pints and 4 pints of freshly frozen plasma were transfused during the operation to compensate an estimated blood loss of 2000 mL. The mother and neonate did not develop complications during their subsequent puerperal and neonatal periods, respectively, and the boy has, thus far, 3 years after birth, exhibited normal neural development. It was only after completion of the cesarean hysterectomy that we were informed that, while undergoing a laparoscopic right ovarian cystectomy, the mother had also been subjected to a combined laparoscopic electromyolysis.

## 3. Discussion

Uterine rupture during pregnancy can be life threatening and may result in serious maternal and fetal complications including death. Such ruptures can be scarred or unscarred, id est with or without precedence of uterine surgery involving myometrial incision, as in the cases of cesarean deliveries or myomectomies.

Most uterine ruptures occur in scarred uteruses. A rupture of an unscarred uterus is rare (4.54 per 1,00,000 births or, approximately, 1 in 22,000 births), but its frequency is increasing.^[10]^ A study in the Netherlands identified the incidence of uterine rupture in unscarred and scarred uteruses to be 0.7 and 5.1 per 10,000 births, respectively, with the ruptures of unscarred uteruses accounting for 13% of all the ruptures.^[11]^

The incidence of a uterine rupture after a laparoscopic myomectomy has been reported to be lower than 1%,^[3]^ whereas, after myolysis, it remains poorly characterized.

If we classify uterine rupture differently, a uterine rupture may be complete or incomplete. A complete rupture is usually more serious, potentially life threatening for both the fetus and the mother,^[4]^ and antepartum pain is a major indication of its presence.^[12]^

Ruptures may display diverse clinical presentations depending on their location in the uterus and the type of labor analgesia used, thereby rendering their accurate diagnosis particularly challenging. For example, a rupture involving the posterior uterus or parametrium in a patient under epidural anesthesia may result in pronounced vital sign changes, but minimal patient discomfort and no vaginal bleeding are to be expected. In contrast, a rupture involving the upper vaginal wall and cervix may present with heavy vaginal bleeding, whereas a rupture involving the bladder may present with a sudden onset of hematuria.^[13]^ In addition, bradycardia, sometimes preceded by variable or late deceleration, is the most common clinical manifestation of uterine rupture. Nevertheless, no fetal heart rate pattern is a pathognomonic sign of rupture, and fetal heart rate changes are insufficient predictors of the presence or the absence of a rupture.^[14]^ Ultrasound imaging of the abdomen may identify whether a defect in the uterine wall or another pathology underlies the patient’s symptoms.^[15,16]^

In the present case, the observed myometrial wall defect extended from the anterior to the posterior wall via the uterine fundus and, from it, to the left uterine wall. The patient’s vital signs were stable and her discomfort was minimal. Fetal monitoring showed an intermittent variable deceleration, which is not considered to be a serious fetal heart rate abnormality.

Uterine ruptures are typically diagnosed from the identification of a complete disruption of all uterine layers, accompanied by active bleeding and hemoperitoneum during laparotomy.^[17]^ A preoperative, provisional diagnosis of rupture is important even if fetal heart rate abnormalities are often a sufficient indication of the need for urgent delivery.

Uterine rupture may bear multiple adverse and interrelated consequences for the mother, including sequelae of bladder laceration, severe hemorrhage, hysterectomy, and death. Perinatal complications include death or serious morbidity by dint of prolonged intrauterine hypoxia. The frequency of these consequences depends on factors such as the location and the size of the rupture (e.g., a lateral rupture results in higher morbidity than an anterior 1), as well as the speed of intervention.^[18–20]^

The reported perinatal death rate associated with uterine rupture ranges from 5% to 26%.^[9,21]^ Death occurs more frequently in cases of placental separation and/or fetal extrusion.^[21,22]^ In a population-based study in Norway, only 11.9% of the infants were born healthy in the presence of a placental separation and/or an infant extrusion, whereas in the absence of both conditions, this percentage rose to 61.9%.^[21]^

Hypoxic ischemic encephalopathy, severe asphyxia, and fetal death have been reported in cases in which the rupture is so extended that the head of the fetus protrudes, as did the protruded amniotic sac with fetal head described here.^[5,6]^

Surgical intervention of a uterine rupture within 10 to 37 minutes is of paramount importance to minimize the risk of permanent perinatal fetal injury. Even so, the fetus may suffer severe hypoxia and metabolic acidosis or serious neonatal consequences.^[7–9]^ However, when the fetus or the placenta extrude from the uterine wall, irreversible fetal damage alongside major neonatal complications are likely to have already occurred; therefore, rapid intervention is not a panacea for all possible fetal abnormalities. In fact, the severity of the outcome largely scales with the degree of placental separation following uterine rupture, which ranges from no separation to total placental abruption, yet a partial separation may increase over time. In the most serious cases, an acute total placental separation as an immediate consequence of a rupture requires almost immediate delivery of the fetus to reduce the risk of fetal death or hypoxic-ischemic encephalopathy. Taking such immediate action is nearly impossible in most delivery units.

In the current case, an emergency cesarean section was performed 4 hours after the uterine rupture had been detected by MRI. During that time, the fetal heart rate pattern displayed intermittent variable deceleration, yet a neonate was delivered without fetal compromise.

In similar cases of remote from term andantepartum uterine dehiscence, successful outcomes by means of expectant management, close monitoring, and early delivery have been reported.^[23–25]^

Fetal compromise is an unlikely event when the uteroplacental blood flow is sufficiently maintained. This case demonstrates the importance of urgent delivery before, or immediately after, the onset of fetal heart rate abnormalities (suggesting the presence of placental abruption or extrusion) rather than upon uterine rupture onset. Although, fortunately, both mother and fetus had an uneventful neonatal and puerperal course in this case, if uterine rupture is strongly suspected clinically, clinician vigilance and urgent delivery is required.

Studies before 1978 reported relatively high rates of fetal mortality related to uterine rupture.^[26]^ In a review of 33 studies by Schrinsky and Benson, 960 cases of uterine rupture led to 620 infant deaths, resulting in a perinatal mortality rate of 65%.^[26]^ In addition, Landon et al reported a perinatal mortality rate due to uterine rupture of 2% (2 out of 124 cases) among 19 academic centers in the United States. These studies have shown that the incidence of perinatal death related to uterine rupture is diminishing in the modern era.^[27,28]^

Uterine rupture can cause severe bleeding, especially when a hysterectomy is needed. Cowan et al^[28]^ demonstrated that, among 5 patients who developed a uterine rupture, the mean blood loss had been 1500 mL and substantial enough to be symptomatic in 3 of them (60%). In another study, 25% (7 out of 28 women) of women who underwent uterine rupture during trial of labor after cesarean section received blood transfusion.^[29]^ In the present study, the estimated blood loss was 2000 mL, and 5 pints of packed red blood cells and 4 pints of freshly frozen plasma needed to be transfused during the operation.

The decision to repair the defect or perform hysterectomy is based on a combination of factors including the extent of uterine damage from the rupture, the patient’s desire for a future pregnancy, their intraoperative anesthetic and hemodynamic stability, and the surgeon’s experience in repairing a complicated rupture. Studies involving single cases and small cohorts have reported a cesarean hysterectomy in 34% to 70% of patients with a uterine rupture.^[17]^

In the present case, cesarean hysterectomy was performed owing to the multiple myometrial wall defects and the extensive thinning identified, as well as the persistence of bleeding from the flaccid uterus after the reconstruction of the uterine wall.

Maternal death due to uterine rupture occurs nowadays at a rate of 0% to 1% in developed countries, but the mortality rates in developing countries remain at 5% to 10%.^[30,31]^ As contemporary medical facilities become increasingly available, the latter rate is likely to converge to the value of the former.

In the present case, while initially, no notable abnormalities were identified by ultrasound and fetal monitoring, an MRI scan and a repeat ultrasound examination revealed a uterine rupture and a protruding amniotic sac with fetal head. We assume that the uterine rupture occurred shortly before the MRI.

This report emphasizes the need for careful consideration of certain clinical implications. For women who wish to conceive in the future, myomectomy or electromyolysis is not recommended when they are accompanied by asymptomatic fibroids. This is because, in the event of a uterine rupture occurring during a subsequent pregnancy, a cesarean section will be needed. Accordingly, even in the presence of small fibroids, myolysis may lead to detrimental sequelae. Thus, it is advisable that surgeons inform patients about the possibility of a uterine rupture during a subsequent pregnancy before performing uterine surgery, and the patients who conceive should inform their doctor about their operation history in detail. Previous uterine surgery should be carefully documented, and, in such cases, the possibility of uterine rupture should always be appreciated especially if a pregnant woman has abdominal pain.

## Author contributions

**Conceptualization:** Seung-Woo Yang, Myeong Ja Jeong, Myounghwan Kim.

**Data curation:** Seung-Woo Yang, Sang-Hee Yoon, Jin-Sung Yuk, Myeong Ja Jeong, Myounghwan Kim.

**Writing – original draft:** Seung-Woo Yang, Sang-Hee Yoon, Jin-Sung Yuk, Kyoung-Chul Chun, Myeong Ja Jeong, Myounghwan Kim.

**Writing – review & editing:** Myounghwan Kim.
